# Impact of bladder volume precision control on setup errors and dosimetry in intensity-modulated radiation therapy for cervical cancer

**DOI:** 10.1186/s43046-026-00343-0

**Published:** 2026-03-16

**Authors:** Zhijiang Lu, Yajun Li, Dehong Luo, Che Chen, Changjiang Zhang, Hailun Wang

**Affiliations:** First People’s Hospital of Zunyi, Zunyi, China

**Keywords:** Cervical cancer, Intensity-modulated radiation therapy, Bladder volume, Dosimetry

## Abstract

**Background:**

Cervical cancer is the most common gynecologic malignancy and a leading threat to women’s health. Intensity-modulated radiation therapy (IMRT) is a cornerstone treatment, but its success depends on precise patient positioning and consistent bladder distension to minimize setup errors and organ motion. This study evaluated the impact of bladder volume variation on setup errors during IMRT and the accuracy and clinical utility of a bladder volume scanner (BVS) for bladder volume management.

**Methods:**

We conducted a prospective, allocated comparative study of 62 cervical cancer patients treated with IMRT between April 2021 and August 2022. Patients were allocated to a BVS group (*n* = 31) that used a bladder scanner to maintain a target bladder volume of 250–350 mL (± 30 mL) or to a control group (*n* = 31) that relied on a strong urge to urinate for bladder filling. We compared bladder volume consistency, setup errors in left-right (X), anterior-posterior (Y), and superior-inferior (Z) directions, homogeneity and conformity indices, and the volumes of bladder, rectum, and small intestine receiving 45 Gy between groups. We also analyzed the correlation between bladder volume measured by BVS and by computed tomography (CT).

**Results:**

The BVS group had significantly more consistent bladder volumes and significantly smaller setup errors in all directions than the control group. The median (interquartile range, IQR) setup errors in the BVS vs. control group were 1.0 (0.8–1.5) vs. 1.7 (1.2–2.5) mm in the X-axis, 0.4 (0.2–1.1) vs. 1.9 (1.5-3.0) mm in the Y-axis, and 1.1 (0.9–1.3) vs. 2.8 (2.0-3.5) mm in the Z-axis, respectively (all *P* < 0.01). The BVS group also showed improved dosimetry, with a better homogeneity index (1.01 vs. 1.11) and conformity index (0.98 vs. 0.94), and significantly lower bladder, rectum, and small intestine volumes receiving 45 Gy (24.6% vs. 29.1%, 19.7% vs. 25.3%, and 17.4% vs. 18.1%, respectively; all *P* < 0.01). Bladder volumes measured by the scanner were strongly correlated with CT-derived volumes (*R* = 0.977).

**Conclusions:**

Precise bladder volume control using a BVS leads to more reproducible patient positioning, as evidenced by significantly reduced bony anatomy-based setup errors, and resulted in significantly reduced radiation exposure to adjacent organs. The strong correlation between BVS and CT measurements confirms the device’s reliability. By ensuring a reproducible daily anatomical setup, BVS-guided bladder management improves the precision of IMRT delivery. This approach mitigates a key source of treatment uncertainty and establishes a foundation for potentially safer margin reduction or dose escalation strategies in the future. The bladder scanner was highly accurate and reproducible, improving target delineation and potentially allowing safer dose escalation.

## Introduction

Cervical cancer, also known as uterine cervical cancer, is a malignant tumor of the female reproductive tract that occurs in the cervix. The most common type is squamous cell carcinoma. The main cause of the tumor is infection by human papillomavirus (HPV), especially types 16 and 18 [1]. Cervical cancer ranks fourth among female malignant tumors globally, following breast cancer, colorectal cancer, and lung cancer [2]. With a 5-year survival rate of less than 60% [3], cervical cancer poses a significant threat to women’s lives and health. Previous studies on cervical cancer have shown that for stage IIB and above, radiotherapy is the first choice; intensity-modulated radiotherapy (IMRT) has significant advantages in treating cervical cancer and offers a clear protective advantage for normal tissues [4–6]. Although IMRT has improved the conformity of the target area to over 70% through dose sculpting techniques [7], the dynamic deformation of pelvic organs remains the main bottleneck for dose delivery accuracy. Fluctuations in bladder volume are a key contributor to this problem, leading to substantial cervical displacement and necessitating large planning target volume (PTV) margins in clinical practice to ensure coverage [8]. This geometric expansion inevitably results in increased radiation dose to adjacent organs at risk [3].This finding highlights a fundamental limitation of the traditional geometric approach: it passively compensates for organ movement by expanding the treatment margin, but does not address the underlying cause—the variable anatomical state driven by factors like bladder filling. Consequently, larger margins are used to ensure target coverage, which inevitably increases radiation exposure to healthy tissues.

Currently, the international radiotherapy community faces a dual dilemma in organ motion management.On the one hand, subjective bladder control strategies (such as quantitative drinking) are limited by individual metabolic differences, post-radiotherapy changes in bladder tissue compliance (e.g., due to fibrosis), and neurogenic micturition reflex disorders [9]. Subjective bladder-filling protocols are known to have poor inter-fraction reproducibility, making it difficult for patients to consistently achieve a comparable bladder volume across treatment sessions. This variability can lead to significant displacement of the target volume [10], as demonstrated by studies showing that bladder filling status is a major determinant of cervical and uterine motion, with displacements large enough to compromise PTV coverage [11]. On the other hand, existing image-guided techniques (such as cone-beam computed tomography [CBCT] and ultrasound) can capture setup errors but cannot fundamentally suppress the biological driving factors of organ motion. For example, although ultrasound guidance can monitor bladder volume in real time, its operation depends on the technician’s experience, may disrupt the treatment flow, and has insufficient spatial resolution for deep target areas [4]. Magnetic resonance imaging (MRI) simulation provides soft tissue contrast but is too costly and time-consuming to meet the daily needs of radiotherapy [12]. This imbalance between “passive correction” and “active regulation” leads to a zero-sum game between target coverage and organ-at-risk protection in radiotherapy for cervical cancer.

## Materials and methods

### Study subjects

This study included 62 patients with cervical cancer who were consecutively admitted to the First People’s Hospital of Zunyi, from April 2021 to August 2022, with ages ranging from 33 to 74 years (median age, 53 years). All patients had pathologically confirmed cervical cancer with FIGO stages IIB to IVA. Patients with severe heart and lung dysfunction, bone marrow suppression (white blood cell count < 3.0 × 10^9^/L, platelet count < 80 × 10^9^/L), active infection, or other contraindications to radiotherapy were excluded. All patients were receiving radical concurrent chemoradiotherapy for the first time. The study was approved by the hospital’s ethics committee (approval number, Lunshen (2021)-1-11), and all patients signed informed consent forms. Assignment to the study groups was performed using a predetermined alternation protocol. Consecutively enrolled patients were allocated to either the BVS group or the control group in a 1:1 ratio following the order of enrollment. Specifically, the first eligible patient was assigned to the BVS group, the second to the control group, the third to the BVS group, and this sequence continued throughout the recruitment period. This allocation process was managed by a research coordinator. The attending radiation oncologists were not involved in the group assignment decision and were only informed of the allocated protocol after the patient was enrolled. This method was employed to minimize selection bias and to prospectively form two comparable cohorts for comparison. Namely, the control group (*n* = 31) used the self-bladder control method (drinking 500 mL of water and holding urine until a strong urge to urinate), while the study group (*n* = 31) used a portable ultrasound bladder volume scanner (model PBS V4.2, Mianyang Meike Electronic Equipment Co., Ltd., China) to precisely control bladder volume (target volume: 250–350 mL, allowing a deviation of ± 30 mL). The patients completed standardized bladder volume control before CT positioning, and it was ensured that the bladder volume was consistent with the positioning state through ultrasound re-measurement or subjective urine sensation assessment before each treatment. Dynamic dose optimization was ultimately implemented through IMRT.

### Exclusion and inclusion criteria

The exclusion criteria were as follows: (1) insufficient treatment compliance; (2) severe comorbidities, such as uncontrolled heart failure (NYHA III–IV), Child–Pugh C liver function, chronic kidney disease (CKD 4–5), or other systemic diseases that may interfere with treatment safety; (3) risk of loss to follow-up, i.e., inability to complete at least 6 months of standardized follow-up or missing key clinical data; (4) pregnancy or lactation; (5) cognitive impairment, defined as the presence of severe mental illness or cognitive impairment, and inability to cooperate with the treatment process or sign an informed consent form.

The inclusion criteria were as follows: (1) diagnostic certainty: pathologically confirmed cervical cancer and imaging (MRI/CT) consistent with FIGO staging criteria; (2) indications for treatment: meeting the indications for radical or adjuvant IMRT as recommended by the International Gynecological Cancer Society (IGCS) guidelines; (3) feasibility of full treatment: receiving full radiotherapy and standardized follow-up at this hospital; (4) ethics compliance: voluntarily signing written informed consent for radiotherapy and clinical research; (5) data integrity: complete entry of baseline data, treatment parameters, and follow-up records into the electronic medical record system, with traceable verification.

### Methods

**Fig. 1:**
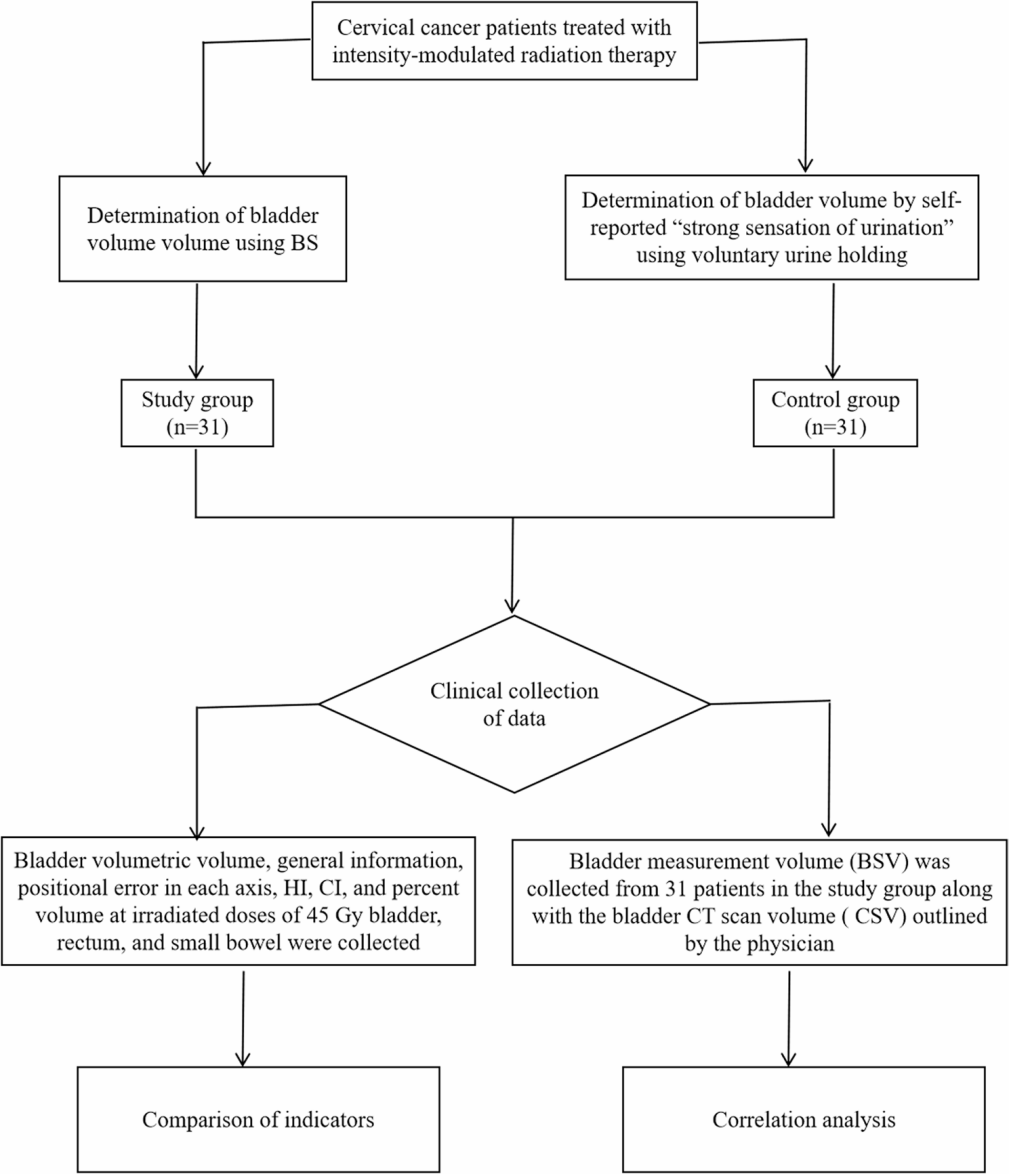
Research flowchart

This was a prospective, randomized controlled study. The study design is shown in Fig. 1. All patients underwent a standardized process as follows: (1) CT simulation positioning and image acquisition: Patients completed CT scan preparation in a fasting state (emptying the bladder 1 h before positioning and drinking 500–1000 mL of water), lying in the supine position with arms crossed over the forehead and legs naturally extended and fixed on a carbon fiber body board (three-dimensional laser positioning system marked body surface lead reference points, i.e., the left and right iliac crests and the midline of the pubic symphysis), and patients underwent pelvic enhanced scanning with a Philips large-bore CT (slice thickness, 5 mm). Images were transmitted to the Elekta Monaco 5.11 radiotherapy planning system via the DICOM protocol. (2) Standardized management of bladder volume: The research group used the Mianyang Meike PBS V4.2 ultrasound bladder volume measurement instrument (with a fixed deputy chief nurse as the operator) to quantitatively control the degree of bladder filling.The standardized treatment range of 250–350 mL was chosen to ensure a consistent, comfortable, and clinically effective bladder volume that displaces the small bowel and stabilizes uterine position [8], rather than strictly reproducing the potentially variable simulation volume. The larger intake during planning CT (up to 500 mL) was allowed to assess individual capacity and ensure good image quality. The specific process was as follows: The largest cross-sectional liquid dark area of the bladder was located 2 cm above the pubic symphysis. Then, the probe was vertically pressed against the skin until a slight depression was formed (constant pressure ≤ 5 N), and the bladder volume was measured to reach the preset target range of 250–350 mL. A deviation of ± 30 mL from this range was allowed to accommodate immediate patient comfort. This range was selected based on a balance between achieving consistent organ displacement (as supported by literature [8]) and maintaining patient tolerance during the treatment course. CT scanning was immediately performed after reaching the standard. In the control group, scanning was completed based on the patient’s subjective perception of the urge to urinate (self-reporting a “strong urge to urinate” was considered equivalent to the state at the time of positioning). (3) Target delineation and plan design: A single senior radiation oncologist delineated the clinical target volume (CTV, covering the primary lesion and high-risk lymphatic drainage areas) and organs-at-risk (bladder, rectum, and small intestine) in accordance with the RTOG (Radiation Therapy Oncology Group) consensus guidelines for contouring in cervical cancer radiotherapy [13]. To generate the planning target volume (PTV), a uniform three-dimensional expansion of 10 mm was applied to the CTV for all patients in both the BVS and control groups. This ensured that the PTV-CTV margin was identical between groups, allowing for a direct comparison of dosimetric outcomes. The physicist used the Elekta Monaco system to design a 7-field IMRT plan (6 MV X-rays). The prescribed dose was 50.4 Gy delivered in 28 fractions of 1.8 Gy each, 5 fractions per week. Dose constraints included: PTV coverage ≥ 95%, bladder Dmean < 45 Gy, rectum V40 < 35%. (4) Treatment implementation and quality control: For patients in the BVS group, the bladder volume was measured using the PBS V4.2 device prior to each treatment session. The target was to reproduce the patient’s individual bladder volume as established during the simulation CT (within the 250–350 mL range). An operational threshold of ± 10% deviation from this reference volume was predefined. For a typical reference volume of 300 mL, this corresponds to an allowable range of approximately ± 30 mL, aligning with the tolerance used during simulation. If the measured volume fell outside this threshold, a corrective protocol was followed: for volumes exceeding 110% of the reference, the patient was asked to partially void and the volume was re-measured; for volumes below 90%, the patient was asked to drink a small amount of water (approximately 100 mL) and wait 10–15 min before re-measurement. Treatment commenced only after the bladder volume was within the acceptable ± 10% range. Before each radiotherapy session, two-dimensional anterior-posterior (AP) and lateral electronic portal images (EPIs) were acquired using the electronic portal imaging device (EPID). Online positioning verification was performed by registering these EPIs to the corresponding digitally reconstructed radiographs (DRRs) generated from the planning CT scan. The registration was performed based on bony anatomical landmarks (primarily the pelvic bones, including structures such as the pubic symphysis, ischial tuberosities, and sacrum) rather than on soft-tissue or tumor anatomy. This approach was chosen to assess the consistency of the patient’s skeletal setup, which is the foundation for reproducible dose delivery. The translational shifts (setup errors) reported in this study therefore reflect deviations in bony anatomy alignment. The registration was based on bony anatomical landmarks, primarily the pelvic bones (pubic symphysis, ischial tuberosities, and sacrum). The three-dimensional displacement values (left–right, anterior–posterior, superior–inferior) were obtained as translational corrections provided by the grayscale registration algorithm when aligning the EPID images with the corresponding DRRs based on bony anatomy. These values were recorded as the setup errors. Positioning error data were collected three times each in the early, middle, and late stages of treatment (a total of nine times per person). Measurement consistency was evaluated based on the Bland–Altman method. Throughout the process, the medical physics team performed dose verification (γ pass rate standard, 3%/2 mm ≥ 95%) and acute toxicity monitoring (CTCAE v 5.0 standard).

#### Equipment information

The PBS V4.2 Ultrasonic Bladder Volume Measurement Instrument, produced by Mianyang Meike Electronic Equipment Co., Ltd., was used in this study. The core working principle of this device is based on ultrasonic echo distance measurement technology. Namely, it emits high-frequency sound waves (2.5–5.0 MHz) and receives reflected signals from the bladder wall, using an internal algorithm to calculate bladder volume in real time. The device consists of a wide-band curved array ultrasound probe (scanning depth, 15 cm; axial resolution, ≤ 1 mm), an integrated main unit (including a 32 GB flash storage module and Bluetooth/Wi-Fi wireless transmission unit), and a rechargeable lithium battery (with a battery life of ≥ 8 h). It features a 7-inch true-color LCD display (resolution, 800 × 600 pixels) that simultaneously shows multi-plane ultrasound images of the bladder. Measurement data can be output through a thermal printer (50 mm/s) as a paper report or stored as encrypted PDF/CSV format files and can be batch-exported to an external computer via a USB 3.0 interface. The device complies with the ISO 13,485 medical device quality management system standard and has been verified with body phantoms, demonstrating a measurement error of not more than ± 5% (for the 50–1000 mL range), meeting the clinical needs for accurate bladder volume monitoring in radiation therapy. According to the manufacturer’s specifications and phantom verification studies cited in its manual, this device has a reported measurement accuracy of ± 5% for volumes ranging from 50 to 1000 mL.

#### Dose calculation and dosimetric evaluation

All treatment plans were calculated using the Monte Carlo algorithm within the Elekta Monaco treatment planning system (version 5.11) with a calculation grid size of 3 mm, performed on the planning CT scan to account for tissue heterogeneity. Dose-volume histograms (DVHs) were generated for the planning target volume (PTV) and organs at risk (bladder, rectum, small intestine). For the PTV, conformity index (CI) and homogeneity index (HI) were derived from DVH analysis as defined in the Results section. For organs at risk, the volume receiving at least 45 Gy (V45) was calculated as a percentage of the total organ volume to evaluate intermediate-to-high dose exposure related to late toxicity. Final dose distributions and DVH parameters were reviewed and approved by both the treating radiation oncologist and a qualified medical physicist prior to treatment implementation.

### Statistical methods

Data analysis was performed using SPSS 26.0. For normally distributed variables, inter-group comparisons were conducted using Student’s *t* test. Non-normally distributed variables were expressed as the median with interquartile range (M (P25–P75)) and analyzed via the Mann–Whitney *U* test. Categorical variables were compared using the chi-square test (expressed as n (%)). Spearman’s rank correlation analysis was applied to evaluate the accuracy of bladder volume measurements by the PBS V4.2 device in cervical cancer patients undergoing IMRT. Statistical significance was defined as *P* < 0.05. Given the primary aim of comparing two clinical strategies, the analysis focused on inter-group comparisons of summary statistics. A detailed per-fraction correlation between continuous volume changes and setup shifts, while of interest, was not feasible within the scope of the present data collection protocol.

## Results

### Comparison of bladder volumes between the two groups

**Fig. 2:**
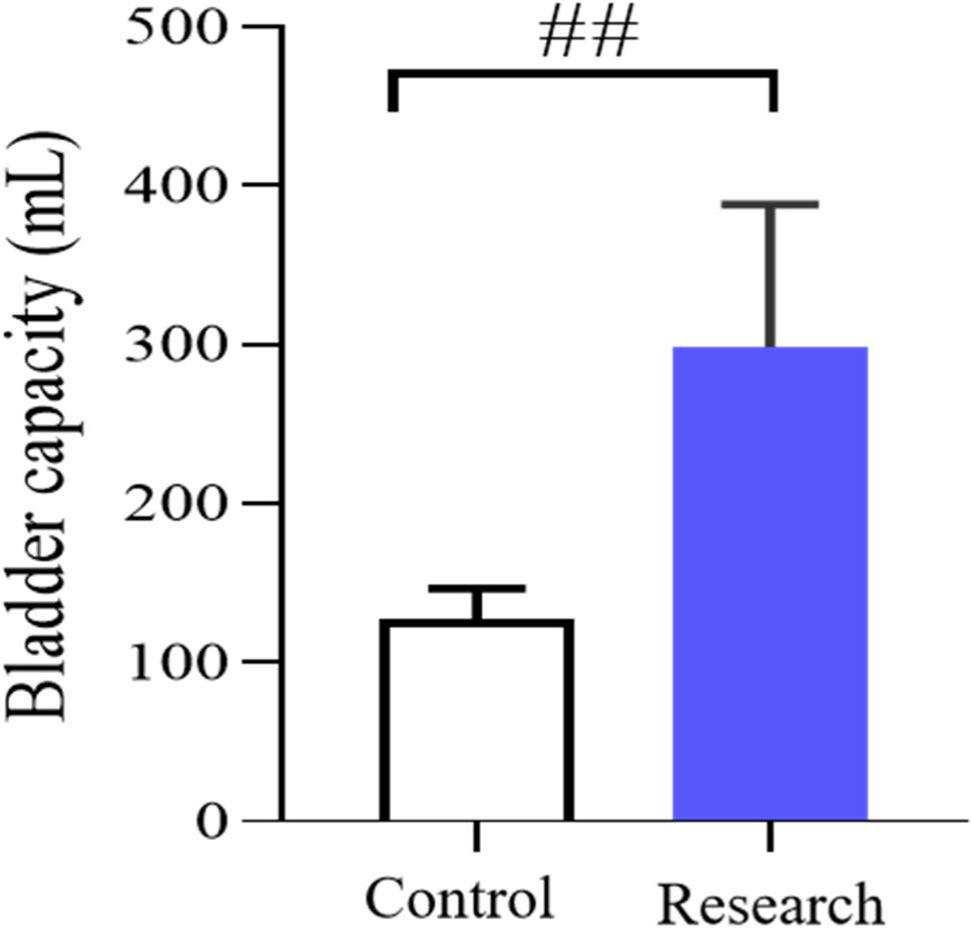
Comparison of bladder volume between the two groups of patients

The bladder volume measured by CT was 298.39 ± 89.89 mL in the study group and 127.23 ± 19.15 mL in the control group. The bladder volume in the study group was significantly greater than that in the control group (*P* < 0.01), as shown in Fig. 2.

### Comparison of general data between the two groups

A total of 62 patients were included in the study and were divided into the study group (*n* = 31) and the control group (*n* = 31) based on two bladder filling methods: one in which bladder volume was ensured to be the same through instrument measurement before treatment, and the other by direct radiotherapy after bladder filling through urinary retention. Statistical analysis revealed no significant differences in the general data between the two groups (*P* > 0.05), as shown in Table 1.

**Table 1:** The comparison of general information between the two groups ($\:\stackrel{-}{\mathbf{x}}\pm{s}$)

Considerations	Research group	Control group	t/χ^2^	*P*
	(*n* = 31)	(*n* = 31)		
Age (years)	52.16 ± 10.57	55.00 ± 8.50	-1.165	0.249
BMI (kg/m^2^)	22.95 ± 2.26	22.70 ± 2.72	0.386	0.701
Diabetes				
Yes	13(41.9%)	6(19.4%)	3.718	0.054
No	18(58.1%)	25(80.6%)		
Hypertension				
Yes	5(16.1%)	8(25.8%)	0.876	0.349
No	26(83.9%)	23(74.2%)		
Tumor differentiation				
Low-polarization	13(41.9%)	14(45.2%)	0.066	0.798
Middle-polarization	18(58.1%)	17(54.8%)		
Tumor Diameter (cm)	5.15 ± 0.30	5.15 ± 0.322	-0.045	0.964
KPS (scores)	81.94 ± 15.02	87.93 ± 11.08	-1.788	0.079

### Comparison of radiotherapy setup errors between the two groups

The target center displacement errors in the X-axis (left–right direction), Y-axis (anterior–posterior), and Z-axis (superior–inferio direction) of the study group were significantly smaller than those of the control group (*P* < 0.01), as shown in Table 2.

**Table 2:** Comparison of radiotherapy placement errors between the two groups of patients [M (P25, P75), mm]

Target area	Research group	Control group	Z	*P*
	(*n* = 31)	(*n* = 31)		
X	1.00(0.77, 1.53)	1.67(1.23, 2.47)	-3.846	< 0.01
Y	0.43(0.23, 1.10)	1.90(1.46, 3)	-5.348	< 0.01
Z	1.10(0.90, 1.33)	2.77(2.00, 3.47)	-4.432	< 0.01

### Comparison of PTV dosimetric parameters between the two groups

The dosimetric parameters of PTV for both groups, including the conformity index (CI) and homogeneity index (HI), were compared. The CI was calculated using the formula: CI = (TV < sub > PIV</sub> )² / (TV × PIV), where TV < sub > PIV</sub > is the volume of the target covered by the prescription isodose, TV is the target volume, and PIV is the total volume of the prescription isodose. A CI value closer to 1 indicates better dose conformity. The HI was calculated as the ratio: *HI = D < sub > 2%</sub> / D < sub > 98%</sub>*, where D < sub > 2%</sub > and D < sub > 98%</sub > are the minimum doses delivered to 2% and 98% of the target volume, respectively. An HI value closer to 1 indicates a more homogeneous dose distribution.

As shown in Table 3, the BVS group demonstrated a significantly higher CI (0.98 ± 0.10 vs. 0.94 ± 0.01, *P* < 0.01) and a significantly lower HI (1.01 ± 0.003 vs. 1.11 ± 0.01, *P* < 0.01) compared to the control group. Both results indicate superior dosimetric quality in the BVS group, with dose distributions being both more conformal and more homogeneous.

**Table 3:** Comparison of PTV dosimetric indexes in the two groups of patients ($\:\stackrel{-}{\mathbf{x}}$± s)

Indicators	Research group	Control group	t	*P*
	(*n* = 31)	(*n* = 31)		
CI	0.98 ± 0.10	0.94 ± 0.01	3.659	< 0.01
HI	1.01 ± 0.003	1.11 ± 0.01	-12.092	< 0.01

### Comparison of the volume percentage of organs-at-risk receiving 45 gy between the two groups

The volume percentages of the bladder and rectum receiving radiation at 45 Gy were significantly lower in the study group than in the control group (*P* < 0.01), as shown in Table 4.

**Table 4:** Comparison of the volume of bladder, rectum, and small intestine at irradiation dose of 45 Gy in the two groups of patients ($\:\stackrel{-}{\mathbf{x}}$± s)

Organ	Research group	Control group	Z	*P*
	(*n* = 31)	(*n* = 31)		
Bladder	24.56 ± 0.79	29.09 ± 1.08	-3.382	< 0.01
Rectum	19.72 ± 0.48	25.34 ± 0.64	-7.024	< 0.01
Small intestine	17.40 ± 0.90	18.08 ± 0.78	-5.71	0.57

### Analysis of the accuracy of bladder volume measurement using the bladder volume scanner (BVS)

**Fig. 3:**
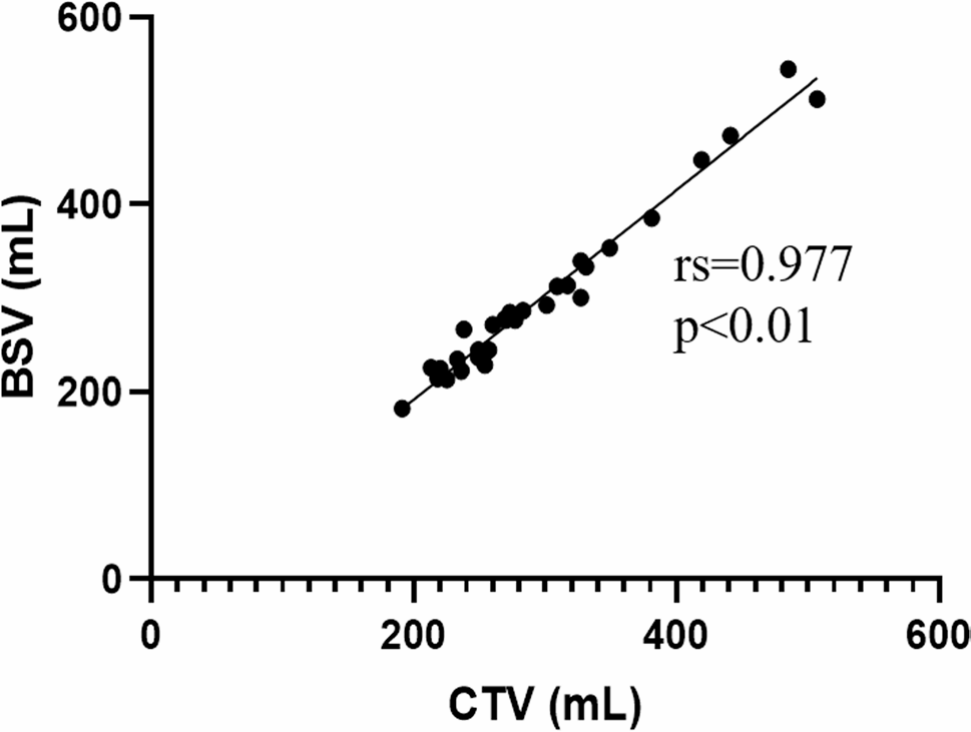
Correlation analysis between BSV and CT-delineated Volume in 31 patients in the study group

A correlation analysis was performed between the BVS-measured volume obtained using a bladder volume measuring device and the bladder CT (CT-delineated volume) outlined by the physician in 31 patients. The results, shown in Fig. 2, revealed a strong linear positive correlation between the two, with the function equation y = 1.1174x − 31.97 and a correlation coefficient (R) of 0.977. This indicates a strong correlation between the two, suggesting that the BVS-measured volume measurement of bladder volume is highly reliable. The results demonstrate that the bladder volume measuring device has high accuracy and can be used to measure bladder filling volume in patients undergoing pelvic radiotherapy, as shown in Table 5; Fig. 3.

**Table 5:** Analysis of the correlation between BVS-measured volume and CT-delineated volume in 31 patients in the study group

	M (P25, P75)	Correlation coefficient	*P*
CT-delineated Volume (ml)	270(238,327)	0.977	< 0.01
BVS-measured volume (ml)	276(234, 333)		

## Discussion

This allocated study provides robust evidence that systematic bladder volume control using a BVS significantly improves the reproducibility of patient setup and resultant dosimetry in cervical cancer IMRT. While the intricate, individual-level correlation between bladder volume fluctuation and immediate target displacement—as quantified in simulation studies [14]—was not the primary analytic endpoint of our clinical protocol, the stark contrast in group-level outcomes offers compelling indirect proof of this relationship.

The clinical implications of breaking this volume-uncertainty link are straightforward and significant. The BVS group’s markedly smaller and more consistent setup errors across all directions (e.g., a 77% reduction in Y-axis median error) and superior dose distributions unequivocally demonstrate that uncontrolled bladder filling is a major contributor to setup uncertainty. When bladder volume is left to subjective sensation (“strong urge”), it introduces a random variable that manifests as increased positional variance. Our protocol, by replacing this variable with an objective, reproducible volume standard, removes a key source of this noise. Consequently, the observed dosimetric benefits—including better target coverage (higher CI), improved dose uniformity (lower HI), and reduced organ-at-risk irradiation—are logically attributed to the treatment of a more consistent anatomical configuration [15]. This represents a practical shift from exclusively reacting to positioning errors via image guidance to proactively minimizing one of their principal causes through volumetric management. Future research incorporating per-fraction imaging and volume tracking is warranted to model the precise “volume-displacement” response curve, which could enable personalized, dynamic PTV margins.

The clinical value of dosimetric optimization is multidimensional. The target area CI improved from 0.94 to 0.98, and the HI decreased from 1.11 to 1.01. The dosimetric improvements observed have direct clinical implications. The superior conformity and homogeneity indices indicate more precise targeting of the prescription dose to the tumor volume, potentially reducing the risks of under-dosing or creating high-dose hotspots within the PTV. More importantly, the significant reduction in V45 for the bladder and rectum is closely associated with a lower predicted probability of late radiation-induced toxicities, such as cystitis and proctitis [16]. These benefits were achieved not by employing more advanced image-guidance or planning algorithms, but through a relatively simple, low-cost intervention—actively stabilizing a key variable anatomical factor (bladder volume). This underscores that optimizing fundamental patient preparation protocols is a highly effective and essential strategy for achieving superior dosimetric outcomes, which are the foundation for reducing clinical toxicity risks.

It is important to acknowledge that pelvic organ configuration, and consequently target position, is influenced by a complex interplay of factors beyond bladder filling alone. Rectal volume variation is a well-established and significant contributor to uterine and cervical displacement While this study focused on standardizing one key variable—bladder volume—future protocols could benefit from a more comprehensive approach that also considers and manages rectal filling status. Our findings on the benefits of bladder volume control, therefore, highlight the potential of proactive anatomical management as a principle. Extending this principle to include multimodal stabilization could further reduce anatomical uncertainty and is a promising direction for next-level precision in pelvic radiotherapy.

The potential obstacles and breakthrough paths for technical promotion deserve further exploration. Although this study showed that the ultrasound bladder volume scanner (BVS) has high reproducibility, its clinical application still faces three main challenges. First, there is the issue of standardizing the operation, as small deviations in probe pressure and scanning depth may lead to cumulative measurement errors. Second, there is the issue of the complexity of multimodal image fusion. Namely, current radiation therapy planning systems (such as Monaco) lack automated registration algorithms, leading to temporal and spatial misalignment between ultrasound data and CT/MRI images. Finally, from a healthcare economics perspective, the cost of equipment procurement and the extended treatment time may affect the willingness of grassroots hospitals to adopt this technology. The key to solving these issues lies in the development of an “intelligent volume control system”—integrating elastic imaging and four-dimensional CT technologies and developing a volume prediction model based on deep learning. This system could predict the optimal bladder volume 24 h before treatment, realizing a “predict–control–verify” closed-loop management approach. Neylon et al. demonstrated that such systems could reduce prostate cancer radiotherapy target displacement errors by 62% [17], providing theoretical support for the technical extension of this study.

With the rapid development of new radiation therapy technologies, precision radiotherapy has become a consensus and developmental direction in current cancer radiotherapy. Precision radiotherapy for pelvic tumors focuses on ensuring bladder fullness and maintaining consistency in bladder volume during fractionated treatments [18, 19]. Traditional methods of bladder filling cannot maintain consistent bladder fullness during each session of radiotherapy, thereby hindering the implementation of precision radiotherapy. Some studies have confirmed the precise application value of bladder volume measurement devices in pelvic radiotherapy [20]. More importantly, this consistent anatomical control has been shown to reduce the need for frequent CBCT verification scans, thereby lowering the additional imaging-related radiation exposure to the patient [21]. In this study, the correlation between the bladder volume measured by the bladder volume meter (BSV) and the bladder volume delineated by the physician from the CT scan (CTV) was analyzed for 31 patients. The results showed a linear positive correlation between the two variables, with the function y = 1.1174x − 31.97 and a correlation coefficient (R) of 0.977. This indicates a strong correlation between the two, confirming the high reliability of BS in measuring bladder volume and further validating its accuracy in monitoring bladder volume prior to IMRT for cervical cancer. Using BS to monitor bladder volume in cervical cancer patients receiving IMRT can maintain consistent bladder fullness, achieving satisfactory results.

Our findings highlight that in addition to technological advancements in imaging and planning, standardizing and optimizing fundamental patient preparation protocols—such as bladder filling—is a critical and effective step toward higher treatment precision. Not only does this paradigm apply to cervical cancer, but it also provides an interdisciplinary solution for radiotherapy in other pelvic tumors, such as prostate cancer and endometrial cancer.

The limitations of this study should also be objectively addressed. First, the single-center design may have introduced selection bias. Second, there was a lack of long-term toxicity follow-up data. Third, due to the prospective nature of data collection, detailed per-fraction bladder volume measurements were not systematically paired with three-dimensional setup errors. This precluded a fine-grained correlation analysis to model the precise quantitative relationship between bladder volume deviation and positional shift, which would further refine clinical margin strategies. Future research should build a multicenter collaborative network, integrate elastic imaging and biomechanical modeling, prospectively capture paired volume and displacement data to establish predictive models, and explore the synergistic effects of volume regulation and adaptive radiotherapy. For example, real-time ultrasound data-based online adaptive planning could dynamically optimize dose distribution in each treatment session, achieving truly “personalized precision radiotherapy.

## Conclusions

This study demonstrates that systematic bladder volume control using a BVS enhances treatment precision by achieving a more reproducible daily anatomical setup. This leads to significantly reduced random setup errors and, consequently, superior dosimetric outcomes including improved target coverage and reduced organ-at-risk doses. Compared to a subjective filling strategy, the BVS-guided approach yielded smaller setup errors (mean reductions of 1.7–2.9 mm across axes), superior dose conformity and homogeneity, and reduced radiation exposure to the bladder, rectum, and small intestine. The BVS itself showed high accuracy and reliability for volume assessment. These findings support the integration of BVS into clinical workflows as a practical and effective method to mitigate uncertainties related to bladder filling, potentially enabling safer treatment delivery and creating a foundation for future strategies like margin reduction or dose escalation. Aslo, it is important to note that the setup errors quantified in this study were based on bony anatomy registration. While this effectively measures the reproducibility of the patient’s skeletal position, it does not account for potential changes in soft-tissue anatomy or tumor morphology (such as shrinkage) that may occur during the treatment course. Our findings demonstrate the benefit of bladder volume control on reducing bony anatomy-based setup uncertainty; however, comprehensive management of tumor motion and deformation may require additional soft-tissue-based image guidance techniques.

## Data Availability

No datasets were generated or analysed during the current study.

## Abbreviations

IMRT: Intensity-Modulated Radiation Therapy
BVS: Bladder Volume Scanner
PTV: Planning Target Volume
CT: CT-delineated volume
HI: Homogeneity Index
V45: Volume receiving 45 Gy radiation dose
KPS: Karnofsky Performance Status
CBCT: Cone-Beam Computed Tomography
MRI: Magnetic Resonance Imaging
RTOG: Radiation Therapy Oncology Group
EPID: Electronic Portal Imaging Device
DIBH: Deep Inspiration Breath Hold
